# Metabolic Control of Smoldering Neuroinflammation

**DOI:** 10.3389/fimmu.2021.705920

**Published:** 2021-06-23

**Authors:** Luca Peruzzotti-Jametti, Cory M. Willis, Regan Hamel, Grzegorz Krzak, Stefano Pluchino

**Affiliations:** Department of Clinical Neurosciences and National Institute for Health Research (NIHR) Biomedical Research Centre, University of Cambridge, Cambridge, United Kingdom

**Keywords:** microglia, macrophages, metabolism, immunometabolism, mitochondria, smoldering inflammation, progressive multiple sclerosis

## Abstract

Compelling evidence exists that patients with chronic neurological conditions, which includes progressive multiple sclerosis, display pathological changes in neural metabolism and mitochondrial function. However, it is unknown if a similar degree of metabolic dysfunction occurs also in non-neural cells in the central nervous system. Specifically, it remains to be clarified (i) the full extent of metabolic changes in tissue-resident microglia and infiltrating macrophages after prolonged neuroinflammation (e.g., at the level of chronic active lesions), and (ii) whether these alterations underlie a unique pathogenic phenotype that is amenable for therapeutic targeting. Herein, we discuss how cell metabolism and mitochondrial function govern the function of chronic active microglia and macrophages brain infiltrates and identify new metabolic targets for therapeutic approaches aimed at reducing smoldering neuroinflammation.

## Introduction

Cellular metabolism is at the foundation of all biological activities (1). While the metabolic processes that support cellular bioenergetics and survival have been extensively studied (2, 3), the role of metabolism in guiding complex cellular functions is yet to be completely understood. Extensive metabolic rewiring occurs in cells to adapt to the local microenvironment in physiological conditions (4), during development (5), and in conditions of disease (6), as cells try to preserve their functions under the shifting availability of energetic substrates.

In this review, we discuss how the regulation of nutrient uptake and consumption is regulated in myeloid cells, when instructed by physiological cues, and as they undergo polarisation in the context of neuroinflammation. Specifically, we highlight how the regulation of their metabolism changes homeostatic cell activities to guide cell activation and signalling in the persistently inflamed central nervous system (CNS).

## Smoldering Neuroinflammation in Progressive MS

Multiple sclerosis (MS) is a chronic inflammatory condition of the CNS that is characterized by demyelination with axonal and neuronal degeneration (7). Most MS patients (~85%) present a relapsing-remitting course (RR), while the remaining ~15% show a primary progressive (PP) disease course characterised by continuous neurological deterioration without definable relapses (8). As the disease evolves, the majority of RR MS patients also advance to a secondary progressive (SP) disease course, usually after 15–20 years from disease onset (9). Despite great successes in the development of therapies for RR MS and disease-modifying therapies that delay the conversion to SP MS (10), progressive MS patients still have limited treatment options (9, 11, 12). Unfortunately, effective treatment of progressive MS remains elusive due to the occurrence of specific degenerative mechanisms that characterize progressive MS, which are distinct from RR MS and are not sufficiently targeted by the approved immunomodulatory compounds (9).

In RR MS, active plaques predominate, and lesions show a diffuse perivascular and parenchymal T cell infiltration that is the substrate of clinical attacks (13). However, as the disease evolves, there is a shift from a T cell mediated adaptive immune response towards an innate immune activation (13, 14). In fact, progressive MS, like many other neurodegenerative CNS diseases [such as Alzheimer’s disease (AD), Parkinson’s disease (PD), and Huntington’s disease], is characterized by a persistent state of CNS inflammation that is driven by myeloid cell activation (8, 15–18).

In progressive MS, myeloid cells are present in the normal appearing white matter (NAWM), in subpial cortical lesions, and, most importantly, in smoldering plaques (13, 19). Smoldering plaques are histopathologically defined as slowly expanding lesions that are characterized by a rim of activated myeloid cells and a slow expansion of the pre-existing plaque edge (19, 20). Here, increased activation of myeloid cells correlates with demyelination and axonal loss, leading to higher clinical disability in patients with progressive MS (8, 14, 18). Indeed, current magnetic resonance imaging (MRI) tools aimed at assessing chronic active and smoldering lesions have emerged as a diagnostic tool to predict secondary disease progression (21, 22), as well as clinical progression in PP MS patients (23).

These data suggest that a slowly expanding, myeloid-mediated, smoldering neuroinflammation is the core feature from which progression starts and evolves in MS. Therefore, understanding the mechanisms underpinning chronic myeloid cell activation in the CNS may hold the promise of identifying new targets to treat and/or delay disease progression (24).

## Myeloid Cells Dynamics in Neuroinflammation

Far from being a homogenous cell population, the cellular makeup of CNS myeloid cells is instead spatially and temporally heterogenous, being under tight regulation by (patho)physiological cues that determine beneficial and/or detrimental immune cell activation (25). Recent single cell technologies have unveiled how the immune landscape of the brain drastically changes with fluctuations in the neuroinflammatory status (26).

In the healthy CNS, immune function is exclusively attributed to parenchymal and extra-parenchymal myeloid cells. Within the brain parenchyma, the most immune-privileged compartment of the CNS, the only resident myeloid cells are microglia (27). Microglia are specialized macrophages that are seeded into the brain from the extra-embryonic yolk sac during embryogenesis (28), and they have key roles in synaptic pruning, phagocytosis, and immune surveillance (29). During early CNS development, microglia are distinguished by several unique transcriptional markers (*Arg1*, *Rrm2*, *Ube2c*, *Cenpa*, *Fabp5*, *Spp1*, *Hmox1*, and *Ms4a7*) associated with cell cycle, phagocytosis, lipid metabolism, and surveillance, which highlights the ongoing maturation of these cells (30). Microglia exhibit dynamic heterogeneity that fluctuates throughout the life of the mouse with the highest diversity occurring in the developmental stage, followed by a decline during adulthood, and increased heterogeneity during CNS diseases (30). In fact, during CNS maturation, healthy adult parenchymal microglia lose their developmental heterogeneity and begin to express “homeostatic” markers (*P2ry12*, *Fcrls*, *C1qa*, *Selplg*, and *Tmem119*) related to lipid metabolism and immune cell interaction (30–32). Interestingly, this transition is seemingly regulated by Maf bZIP transcription factor B (MAFB), which controls myeloid cell differentiation and cellular responses to viral infection (31).

Within the CNS borders, such as the dural meninges, the cellular make-up is mostly dominated by T and B cells with a minority of cells constituting macrophages and monocytes. This diverse and complex immune surveillance network facilitates the interaction between lymphocytes and macrophages at specialized immune hubs located along the dural sinuses (33). Border-associated macrophages (BAMs) [also known as CNS-associated macrophages] localized in the leptomeninges, perivascular space, and choroid plexus, are responsible for immune surveillance, together with a small proportion of other immune cells, such as dendritic cells (DCs) and neutrophils (34). BAMs share similar transcriptional markers with microglia [*Aif1 (*encoding Iba1), *Csf1r*, and *Cx3cr1*] (30, 35) and some transcriptional signatures of developing microglia overlap with BAM clusters (*Ms4a7*, *Ccr1*, and *Mrc1*), possibly suggesting ongoing maturation status (30).

In the context of neuroinflammation, the brain immune landscape drastically changes. In mice affected by experimental autoimmune encephalomyelitis (EAE), an animal model of MS, the CNS is predominantly populated by short-lived infiltrating cells (Ly6C^hi^ and Ly6C^lo^ monocyte-derived cells [MdCs]) and T cells that infiltrate during the acute phase of disease through a “leaky” blood-brain barrier (BBB) (34). Here, BAM cell numbers decrease and their phenotype becomes more homogenous, with nearly all BAMs exclusively expressing MHCII and CD38 (34). At the peak of disease, microglia downregulate homeostatic markers and shift their phenotype towards a pro-inflammatory state whereby they overexpress IFN-γ-responsive genes [*H2* (encoding MHCII) and *Sca1*], which imply increased microglia-T cell interactions. Exclusively to the peak phase of EAE, four sub-populations of disease associated microglia (DAM) emerge (daMG1-4), which are distinguished by their unique expression patterns of chemokines, cytokines, and cysteine proteases (36). Although all four populations are specific to EAE, only three are identified within demyelinating lesions (daMG2-4) and exhibit similar downregulation of homeostatic genes (*P2ry12* ^lo^, *Tmem119* ^lo^, *Md1* ^hi^). DaMG2 upregulates *Cd74*, *Ctsb*, and *Apoe* but proliferate less compared to the daMG3, whereas daMG3 expresses high levels of *Cxcl10*, *Tnf*, and *Ccl4*. Finally, daMG4 overexpress *Ccl5*, *Ctss*, and *Itm2b* (36). Further studies investigating myeloid cells in chronic EAE are required to understand whether these daMG profiles are transient. Nonetheless, similar findings are observed in the brain of patients with MS, where specific DAMs downregulate the expression of homeostatic genes (*TMEM119*, *P2RY12*, and *SLC2A5*) and upregulate *APOE* and *MAFB* in late-active demyelinating lesions (37). These clusters were highly enriched in *CTSD*, *APOC1*, *GPNMB*, *CD74*, *HLA-DRA*, and *HLA-DRB*, which further supports the notion of increased microglial heterogeneity during CNS insult not only in EAE but also in the MS brain (38). Of note, analogous DAM transcriptional changes are also confirmed in the 5xFAD animal model of AD, cuprizone-mediated demyelination, and facial nerve axotomy where downregulation of canonical microglial genes (*P2ry12/13*, *Cx3cr1*, *Tmem119*) is coupled with the upregulation of genes related to phagocytosis and lipid metabolism (*Apoe*, *Lpl*, *Cst7*, *Ctsd*, *Tyrobp*, and *Trem2*) (26).

Understanding how these unique, disease-specific, microglial phenotypes can be targeted to promote a beneficial phenotype that ultimately ameliorates smoldering CNS inflammation is a current research challenge that will certainly uncover new therapeutic avenues.

## Glucose and Glutamine Metabolism

Strong evidence has revealed that changes in the reactive states of macrophages and microglia can be regulated by their cellular metabolism (24). How the unique metabolic environment of the brain regulates the effector function of myeloid cells in health and disease is only now starting to be uncovered.

The CNS has intrinsic high metabolic demands associated with neural activity, as ~20% of the body’s glucose and oxygen is used by the CNS, despite only accounting for 2% of the total body weight (39). Glucose is shuttled from the blood *via* specialized glucose transporters (GLUTs) to provide fuel for cellular functions (40). Despite the high utilization of glucose by the CNS, only a small pool of nutrient reserves is stored as glycogen (41, 42). Therefore, tight regulation of glucose metabolism is critical for brain physiology, as disturbed glucose metabolism may contribute to several neurodegenerative diseases (43, 44).

**Figure 1:**
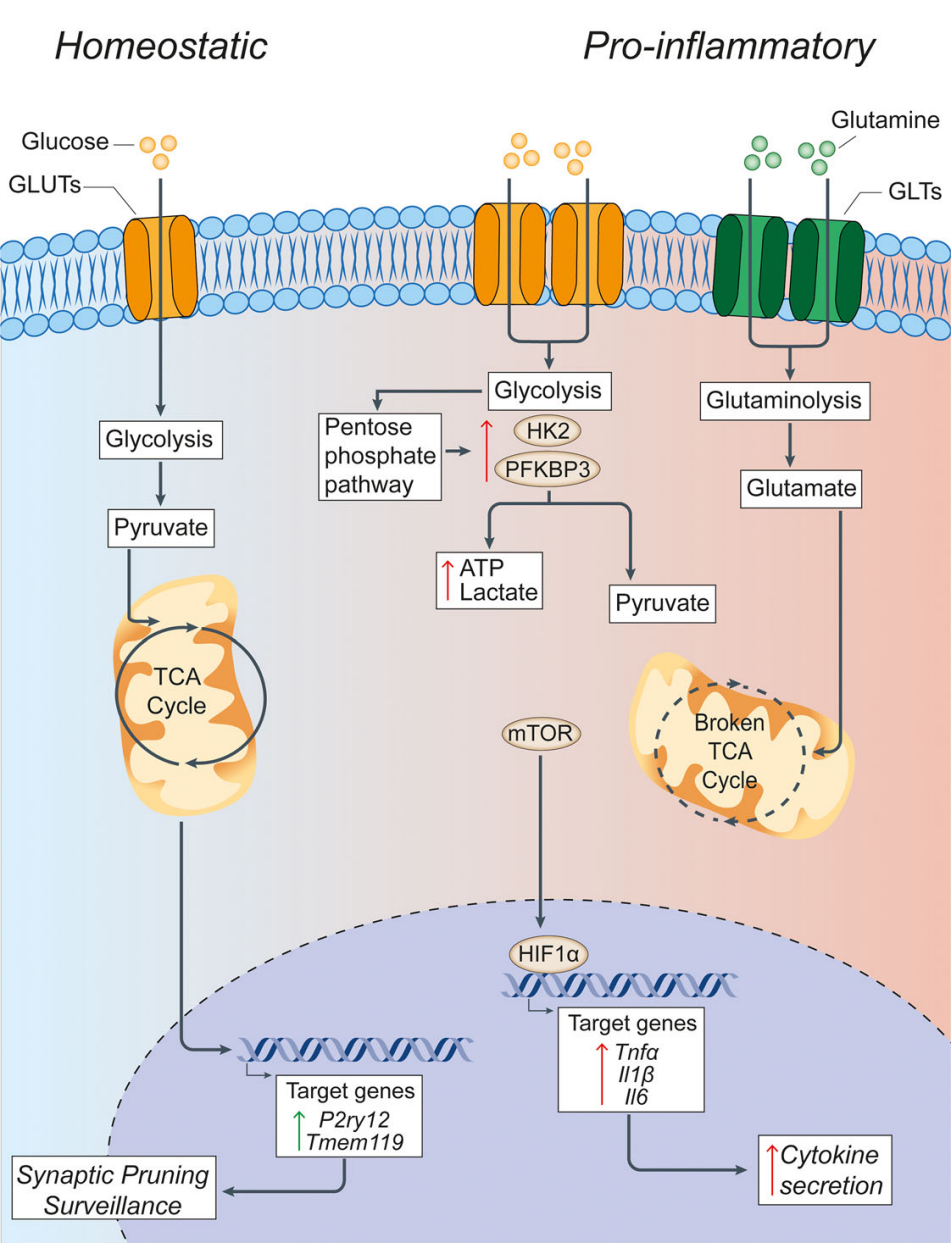
Glucose metabolism in microglia under homeostatic and inflammatory conditions. Under homeostatic conditions, extracellular glucose is transported into microglial cells through specialized glucose transporters, where it is converted into pyruvate through cytoplasmic glycolysis. Pyruvate is then actively transported across the mitochondrial membrane to drive the TCA cycle. The energy and metabolites produced in the TCA cycle can then support the expression of the homeostatic microglial genes *P2ry12* and *Tmem119*, which facilitate microglial functions of synaptic pruning and immune surveillance. In pro-inflammatory conditions, microglia have a broken TCA cycle and increase the expression of membrane transporters to facilitate the uptake of glucose and glutamine, thus driving enhanced glycolysis and glutaminolysis. Glycolysis is supported by increased expression of the rate-limiting enzymes of glycolysis HK2 and PFKBP3. This leads to the increased generation of lactate and ATP to compensate for the broken TCA cycle, and shunting of metabolites into the pentose phosphate pathway. The increased glycolysis is sustained by the activation of nuclear transcription factors HIF1α and mTOR that support the synthesis and production of cytokines for secretion. Green arrows = homeostatic effects. Red arrows = pro-inflammatory effects. GLUT, glucose transporter; TCA, tricarboxylic acid cycle; GLT, glutamate transporter; HK2, hexokinase 2; PFKBP3, 6-phosphofructo-2-kinase/fructose-2,6-biphosphate-3; HIF1α, hypoxia inducible factor 1 α; ATP, adenosine triphosphate; mTOR, mechanistic target of rapamycin; TNFα, tumor necrosis factor alpha; Il1β, interleukin-1β; Il6, interleukin-6.

Microglia require a large amount of energy to perform homeostatic functions. This is accomplished by microglia preferentially utilizing glucose as the main source of metabolic fuel, which is transported into microglia primarily by GLUT1, 3, and 5 (45, 46) to support oxidative metabolism. In oxidative metabolism, glucose is broken down into pyruvate through glycolysis, which is shuttled into the mitochondria where it is utilized by the tricarboxylic acid cycle (TCA) to drive oxidative phosphorylation (OXPHOS); ultimately producing adenosine triphosphate (ATP) (47) (**Figure 1**). Oxidative metabolism is the primary source for energy of microglia under homeostasis, as shown by transcriptomic analysis of *ex vivo* isolated mouse brain microglia, which express the full complement of genes required for both glycolytic and oxidative energy metabolism (48, 49). The ability of microglia to utilize glucose as a primary substrate for energy production has been mainly investigated *in vitro*. Primary rat microglia and the BV2 microglial cell line cultured in the presence of 2-deoxyglucose (2DG), which inhibits hexokinase 2 (HK2) and blocks glycolysis, leads to ATP depletion and cell death (50), indicating a reliance of microglia on glucose utilization for normal functioning. During experimental glucose starvation, primary microglia isolated from CD-1 IGS mice and the BV2 microglial cell line are able to maintain oxidative metabolism using other available substrates (glutamine, lactate, pyruvate, ketone bodies) (51). The reliance of microglia on oxidative metabolism through glucose has been recently confirmed *in vivo* in mice. Using endogenous fluorescence lifetime imaging (FLIM) of intracellular nicotinamide adenine dinucleotide phosphate (NADPH), as well as time-lapse two-photon imaging, microglia have been observed to maintain a glycolytic profile under resting conditions (52). Further, in conditions of aglycemia, microglia switches from glycolysis to glutaminolysis of extracellular glutamine, a major substrate for the generation of both excitatory and inhibitory neurotransmitters (53), in a mechanistic target of rapamycin (mTOR)-dependent manner to maintain OXPHOS and their immune surveillance functions (52). This suggests that microglia display bioenergetic versatility and, like peripheral macrophages, possess the ability to adapt its metabolic pathways to use substrates available in the local environment (51).

The ability of macrophages to shift or reprogram their metabolism to changes in the microenvironment is a key feature that underlies the complex, long-term changes of these cells under inflammatory conditions. In fact, immune cell polarization after inflammatory activation leads to drastic reprogramming of cellular metabolism pathways. A key finding is that macrophages exposed to an inflammatory stimulus shift their metabolism from oxidative metabolism to aerobic glycolysis (54), with a concomitant increase in the enzymatic activity of enzymes involved in glucose metabolism (55) and several transcription factors, including hypoxia-inducible factor-1 alpha (HIF1α) and mTOR (56) (**Figure 1**). This metabolic switch occurs when macrophages are first faced with an immune challenge to support and enable the rapid production of ATP (57) - regardless of the availability of oxygen - which is similar to the Warburg effect described in cancer cells (58). Here, the increased consumption of glucose by activated macrophages leads to the generation of downstream products, such as glucose-6-phosphate and pyruvate, which feed into the pentose phosphate pathway (PPP) and TCA cycle, respectively. The PPP facilitates the synthesis of proteins, nucleotides, and reactive oxygen species (ROS) to support cellular function during immune challenge (59).

Despite the extensive study of metabolic reprogramming in macrophages during inflammation, very little emphasis has yet been placed on assessing metabolism in microglia under neuroinflammatory conditions. Early studies investigating the links between metabolism and microglial activation identified metabolic modifications similar to those observed in peripherally activated macrophages, such as a shift from oxidative metabolism towards a more glycolytic profile after exposure to pro-inflammatory stimuli such as the toll-like receptor (TLR) ligand lipopolysaccharide (LPS). BV2 microglia treated with LPS exhibit increased lactate production and decreased mitochondrial ATP production, which is indicative of a shift to glycolysis (60). Another study confirmed the increased glucose consumption and glycolytic enzyme activity in parallel with increased anaerobic glycolysis and PPP utilization following LPS and IFN-γ treatment (61). Mouse primary microglia treated with IFN-γ result in a metabolic switch towards glycolysis and the retention of iron nanoparticles that is thought to be driven by 6-phosphofructo-2-kinase/fructose-2,6-biphosphate (PFKFB)3, an enzyme involved in glycolysis (62). The treatment of primary microglia with LPS for 24 hours also causes a shift from OXPHOS to glycolysis (63), which is mediated through the activation of the mTOR pathway and leads to enhanced ROS production (64). The metabolic switch is abolished following the addition of the phosphatidylinositol 3’-kinase antagonist LY294002, rapamycin or torin1, which all suppress the phosphorylation of mTOR (64). Further, 2DG treatment of primary mouse microglia in parallel with LPS stimulation inhibits glycolysis with subsequent downregulation of LPS-induced genes (*Il6*, *Il1β*, and *Nos2*) and cytokine production (IL-6 and IL-1β) (65). Recently, primary mouse microglia treated with IL-1β and IFN-γ for 24 hours not only exhibit increased glucose metabolism but also glutamine metabolism through glutaminolysis. Interestingly, within this same study, human microglia-like cells differentiated from pluripotent stem cells treated with LPS for 24 hours exhibit increased *PFKB3* gene expression and increased glycolysis. The use of two-photon FLIM imaging to interrogate the metabolic signatures of individual microglia in acutely prepared mouse hippocampal slices exposed to LPS revealed an increase in aerobic glycolysis in microglia that is blocked by the addition of 2DG (5 mM) (66).

BV2 microglia, and the B6M7 microglial cell line, treated with LPS and IFN-γ exhibit the expected metabolic shift towards enhanced glycolysis and increased gene expression of GLUT1. The inhibition of GLUT1 with STF31 in pro-inflammatory conditions specifically prevents the increase in microglial glucose uptake and attenuates the upregulation of inflammatory cytokines TNF-α, IL-1β, IL-6, and CCL2 *in vitro*, whereas an intraperitoneal injection of STF31 in a mouse model of light-induced retinal degeneration leads to reduced microglia activation and retinal degeneration *in vivo* (46). In BV2 and primary mouse microglia cultured in a hypoxic environment (1% oxygen), HK2, the first rate-limiting enzyme in glycolysis, is increased and correlates with enhanced glycolysis (67). Here, the pharmacological inhibition of HK2 with lonidamine impairs the activation profiles of both BV2 and primary microglia under hypoxia. HK2 blockade prevents ischemic brain injury by repressing microglia mediated neuroinflammation in a rat experimental model of stroke *in vivo* (67).

These studies suggest that under inflammatory conditions, microglia exhibit an increased glycolysis to OXPHOS ratio, similarly to what occurs in peripherally activated macrophages and during the Warburg effect of cancer cells. In summary, these studies strongly indicate that microglial polarization results in significant changes in the preferred metabolic pathway, from oxidative metabolism in homeostasis to a reliance on glycolysis and glutaminolysis in pro-inflammatory states.

Therefore, focusing on small molecules and/or drugs that promote oxidative metabolism over glycolysis will have profound impacts in the way we approach neuroinflammatory and neurodegenerative conditions.

## Lipid Sensing and Signalling in Myeloid Cells

**Figure 2:**
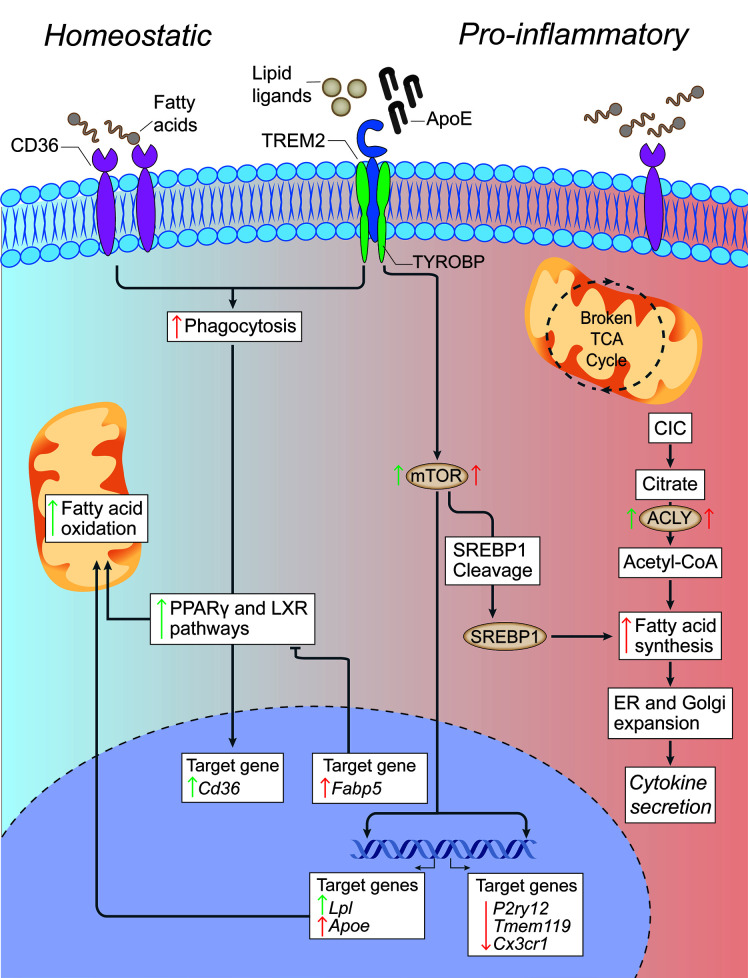
Lipid metabolism in microglia under homeostatic and inflammatory conditions. In microglia, CD36 and TREM2 play a key role in the response to extracellular lipids. CD36 promotes lipid-responsive signalling pathways (like the PPARγ and LXR pathways), which in turn increase FAO and further upregulate *Cd36*. TREM2 activation (via ligands such as ApoE) results in the suppression of homeostatic microglial genes (*P2ry12, Tmem119, and Cx3cr1*), the activation of mTOR signalling, and the upregulation of lipid processing genes (such as *Apoe*, *Lpl*, and *Fabp5*). Despite mTOR increasing both FAS (through the cleavage and activation of SREBP-1) and glycolysis (which are canonically associated with a pro-inflammatory activation of myeloid cells), it appears that the role of TREM2 is to support correct lipid metabolism. In fact,TREM2 deficient microglia show the formation of intracellular cholesterol crystals that activate the inflammasome pathway. On the contrary, the downstream gene *Fabp5* seems to play a key role in determining the pro-inflammatory activation of myeloid cells, possibly via inhibition of PPARγ signalling and FAO. In pro-inflammatory microglia, a broken TCA cycle is coupled with an upregulated mitochondrial CIC, which increases citrate export from the mitochondria to the cytosol, where it is converted into acetyl-CoA for FAS by ACLY. The resultant increase in FAS supports the expansion of the ER and Golgi, and the increased production of pro-inflammatory cytokines. Green arrows = homeostatic effects. Red arrows = pro-inflammatory effects. PPARγ, peroxisome proliferator-activated receptor γ; LXR, liver X receptor; FAO, fatty acid oxidation; mTOR, mechanistic target of rapamycin; FAS, fatty acid synthesis; ACLY, ATP citrate lyase; CIC, citrate carrier; TCA, tricarboxylic acid cycle; SREBP-1, Sterol regulatory element binding protein 1; TYROBP, TYRO protein tyrosine kinase-binding protein; TREM2, Triggering receptor expressed on myeloid cells 2; Lpl, Lipoprotein Lipase; P2ry12, Purinergic Receptor P2Y12; Tmem119, Transmembrane Protein 119, Cx3Cr, C-X3-C Motif Chemokine Receptor 1.

Lipids are fundamental building blocks of cell membranes and myelin in the brain (68–70). In the context of CNS damage, including demyelinating diseases, lipids play a key role in modulating inflammatory responses and contribute to metabolic dysfunction, which is an important aspect of disease pathophysiology (71, 72). In particular, the metabolism of lipids is central to both homeostasis and inflammatory responses in CNS myeloid cells, where it plays vital roles in respiration, activation, inflammatory signalling, migration, and phagocytosis (70, 73). Indeed, recent transcriptomics studies have provided indirect evidence supporting drastic changes in the lipid metabolism of activated microglia, as seen by the upregulation of lipid metabolism genes such as *Trem2, Apoe, Spp1, Cts7, Lpl*, and *Fabp5* under inflammatory conditions (30, 70, 74, 75). However, the role of these genes and pathways is still under investigation, especially in diseases where myelin deposition in the CNS parenchyma can exceed the lipid processing capacity of myeloid cells (**Figure 2**) (73, 76, 77).

The basis of the myeloid cell response to lipids is determined by the carefully regulated composition of phospholipids (PLs) in the cell membrane. PLs are formed from two fatty acids (FAs), a phosphate group, and a glycerol or sphingosine molecule. While they are best known as major components of the cell membrane, PLs are also critical for vesicle formation, apoptosis, and as metabolic intermediates for the production of both pro- and anti-inflammatory molecules (70). Sphingosine containing PLs, also known as sphingolipids, are prominent signalling molecules in the CNS. Sphingosine-1-phosphate (S1P), derived from the phosphorylation of sphingosine, can act as an intracellular intermediate for complex sphingolipid and phosphatidylethanolamine (PE) synthesis or can be released from the cell where it can act *via* autocrine or paracrine signalling through five different G-protein coupled receptors (S1PR_1-5_) (78, 79). The exact role of S1P signalling in myeloid cells remains unclear. The treatment of LPS stimulated mouse primary microglia with the S1P structural analog fingolimod, results in the downregulation of pro‐inflammatory cytokines and the upregulation of brain-derived neurotrophic factor and glial‐derived neurotrophic factor (80). However, *in vitro* evidence suggests that both S1P and fingolimod act *via* astrocytes, rather than myeloid cells or neurons, to suppress chronic neuroinflammation (81, 82). Other data suggests, instead, that signalling through S1PR_1-3_ activates the NF-κB pathway and polarizes microglia towards a pro-inflammatory, amoeboid phenotype *in vitro* and in mouse models of cerebral ischemia (83–86). Ultimately, further research is required to elucidate the myeloid-specific role of S1P signalling in chronic neuroinflammation.

Myeloid cells express specialized scavenger receptors (SCARs) that sense and uptake extracellular lipids, including FAs. The class-B SCAR CD36, also known as FA translocase, is a phagocytic receptor that is widely expressed on microglia and peripheral myeloid cells to facilitate long chain FA uptake and low-density lipoprotein binding (87). Under demyelinating neuroinflammatory conditions, such as MS, CD36 is necessary for the phagocytosis of myelin debris (88, 89). Here, myelin internalization promotes anti-inflammatory lipid-responsive signalling pathways, like the peroxisome proliferator-activated receptor-γ (PPARγ) pathway, which in turn upregulates CD36 (88, 90, 91). This further supports the notion that CD36 serves an anti-inflammatory role as pro-inflammatory microglia have been demonstrated to downregulate CD36 *in vivo* (91) and the *in vitro* inhibition of CD36 in microglia and bone marrow-derived macrophages (BMDMs) promotes inflammation while reducing anti-inflammatory signalling pathways [e.g., PPARγ and liver X receptor] (88).

Another extracellular lipid sensing molecule with implications for chronic neuroinflammation is triggering receptor expressed on myeloid cells 2 (TREM2). TREM2 is a microglia-specific transmembrane receptor with several proposed ligands including ApoE (92), anionic or zwitterionic lipids, PL (93), PE, and phosphatidylserine, which become exposed on the cell surface during apoptosis (94). The binding of TREM2 to extracellular ligands results in the suppression of homeostatic microglial genes and a shift towards an activated phagocytic state (74, 75). It also leads to the activation of mTOR signalling, a pathway that has critical implications for both glycolysis (95) and lipid metabolism (95). In fact, TREM2 has emerged as an innate immune receptor that impacts microglia metabolism through the basic activation of mTOR signalling, which supports long-term cell trophism, survival, growth, and proliferation rather than drastic metabolic reprogramming (95). A recent study used cell type-specific lipidomics to demonstrate that TREM2 is not necessary for myelin uptake by microglia, rather it is required for the upregulation of lipid processing genes involved in lysosome function, cholesterol transport, and cholesterol metabolism, such as *Apoe* and *Lpl* (96, 97). Global TREM2 deficiency hinders the efflux of cholesterol from microglia *in vitro* and *in vivo* and enhances the neurotoxic effect of cuprizone in mouse models of chronic demyelination (96).

Therefore, defective lipid metabolism in TREM2 deficient microglia could result in the accumulation of intracellular cholesterol crystals that damage lysosomes and activate the inflammasome pathway (76).

Understanding the link between lipid sensing, uptake, and intracellular metabolism is therefore key in identifying further targets for therapeutic approaches aimed at resolving chronic inflammation.

## Lipid Metabolism

FAs are transported into mitochondria and used to fuel mitochondrial OXPHOS, a process known as fatty acid β-oxidation (FAO) (**Figure 2**). Changes in FAO in response to inflammatory mediators has been well characterized in peripheral macrophages (98), and more recent studies in microglia have drawn many parallels between these myeloid cells. In peripheral macrophages *in vivo*, alternative activation *via* IL-4 increases FAO through PPARγ signalling (99). Alternatively activated microglia also increase FAO (61, 100), but the involvement of PPARγ signalling has yet to be confirmed. Most importantly, increasing FAO reduces the response to inflammatory perturbations such as LPS in SIM-A9 mouse microglia cells (101), while the inhibition of FAO in human macrophage-differentiated THP-1 monocytic cells (102) and mice microglia *in vitro* and *in vivo* (103) has the opposite effect. Therefore, FAO positively regulates anti-inflammatory responses possibly by minimizing FA metabolites that cause endoplasmic reticulum (ER) stress and act as precursors of pro-inflammatory molecules (70). In line with this, a deficiency in lipoprotein lipase (LPL), a catalyst for the release of FAs that is required for FAO in microglia, causes a shift in microglia metabolism towards glycolysis and increased pro-inflammatory activation (100).

In addition to LPL, lipid metabolism for processes such as FAO can be facilitated through fatty acid binding proteins (FABPs). FABPs are a family of 14-15 kilodalton (kDa) lipid chaperones that reversibly bind hydrophobic molecules, including FAs, and transport them to specific nuclear compartments (104). In homeostasis and activation, peripheral myeloid cells express the FABP isoforms FABP4 and FABP5 (105). Microglia express FABP5 only during development or upon activation (30), suggesting a specific role for FABP5 in activated microglia, which has yet to be discerned. In another type of immune cell, regulatory T cells (Treg), FABP5 loss of function results in decreased OXPHOS and impaired lipid metabolism, ultimately increasing Treg IL-10 production and promoting Treg immunosuppressive activity (106). Furthermore, FABP5 inhibition in CD4^+^ T cells increases PPARγ expression and skews T cell differentiation away from effector T cells (e.g., Th1, Th17) and towards Tregs *in vitro* (107). The same study found that systemic FABP5 inhibition reduces inflammation and improves clinical scores in mouse models of EAE (107). In FABP5 knock out BMDMs, stimulation with inflammatory (LPS and IFN-γ) or anti-inflammatory (IL-4) mediators results in significantly higher expression of anti-inflammatory factors (105, 108). These findings suggest that loss of FABP5 function promotes anti-inflammatory responses in macrophages. Thus, while little is known about the role of FABP5 in microglia, it represents an interesting target that could be manipulated to alter PPARγ signalling and lipid metabolism to reduce chronic neuroinflammation.

Fatty acid synthesis (FAS) is the generation of FAs from the breakdown of the metabolite acetyl-CoA and co-factor NADPH by fatty acid synthases and acetyl-CoA carboxylase in the cytoplasm. Acetyl-CoA is generated from citrate *via* the cytoplasmic enzyme, ATP citrate lyase (ACLY), which is activated in inflammatory macrophages (109). Of note, the role of some of these players can be ambivalent, as IL-4 stimulation of macrophages activates Akt-mTORC1 pathway to phosphorylate and activate ACLY, leading to increased histone acetylation and the upregulation of a subset of M2 genes (110). Myeloid cells challenged with LPS increase FAS through a combination of metabolic and transcriptional pathways. Metabolically, *in vitro* macrophages isolated from histiocytoma and treated with LPS were shown to upregulate mitochondrial citrate carrier (CIC), which exports citrate to the cytosol where it is converted into acetyl-CoA, which is then available for FAS (111). It is also known that LPS increases glycolysis in macrophages, driving flux through the PPP which increases the availability of NADPH for FAS (112, 113). Transcriptionally, LPS activation has been demonstrated to activate mTOR signalling in primary rat microglia and mouse N9 microglia cell lines (114). mTOR activation has been widely shown to increase FAS through the cleavage and activation of sterol regulatory element-binding protein-1, the transcriptional regulator of lipogenesis (112, 113).

Together, the resultant increase in FAS supports the expansion of the ER and Golgi, allowing for increased production of pro-inflammatory cytokines such as IL-6, TNFα, and IL-12 (115). The disruption of FAS reduces both ER and Golgi expansion and pro-inflammatory cytokine secretion in DCs (115), but these findings have yet to be confirmed in myeloid cells.

The emerging field of lipidomics, the improvement of complimentary high throughput techniques, and additional experimental work aimed at assessing the fate of lipids in intracellular organelles (e.g., mitochondria) will reveal exciting roles for lipid metabolism in regulating myeloid cell function and, ultimately, chronic neuroinflammation.

## Mitochondrial Dynamics in Myeloid Cells

Mitochondrial dynamics in tissues, including the CNS and the immune system, are regulated by complex mechanisms (116, 117). Far from being isolated organelles inside cells, mitochondria participate in an active network that is regulated by the local events of fission and fusion, as well as a global control through cellular signalling and metabolic pathways (118).

Fission is mainly controlled by the GTPase dynamin-related protein 1 (DRP1), which is further regulated by several adaptor proteins, such as the mitochondrial fission factor, as well as the mitochondrial dynamics proteins of 49 and 51 kDa and the mitochondrial fission 1 protein (119). DRP1 functions by assembling into oligomeric spirals that constrict and cut the mitochondrion apart by working in concert with dynamin-2 (120). DRP1 activity is further controlled by post translational modifications whereby phosphorylation of serine (Ser) residues Ser 638 or Ser 616 blocks or enhances mitochondrial fission, respectively (121, 122). Fusion is a two-step process that is regulated by the dynamin-like GTPases mitofusin 1/2 on the outer mitochondrial membrane and optic atrophy 1 (OPA1) on the inner mitochondrial membrane. Long forms (L-OPA1) are proteolytically cleaved by peptidases to generate short forms (S-OPA1) to balance fusion (123). Given its location within the inner mitochondrial membrane, OPA1 plays a key role in maintaining cristae morphology, mitochondrial DNA (mtDNA), and supercomplex assembly (124). It is this fine balance between fission and fusion events that regulates key cellular processes, including mitophagy, mitochondrial transport, calcium homeostasis, and mitosis/apoptosis, thus modifying cell metabolic states *via* a bidirectional cross talk (125).

**Figure 3:**
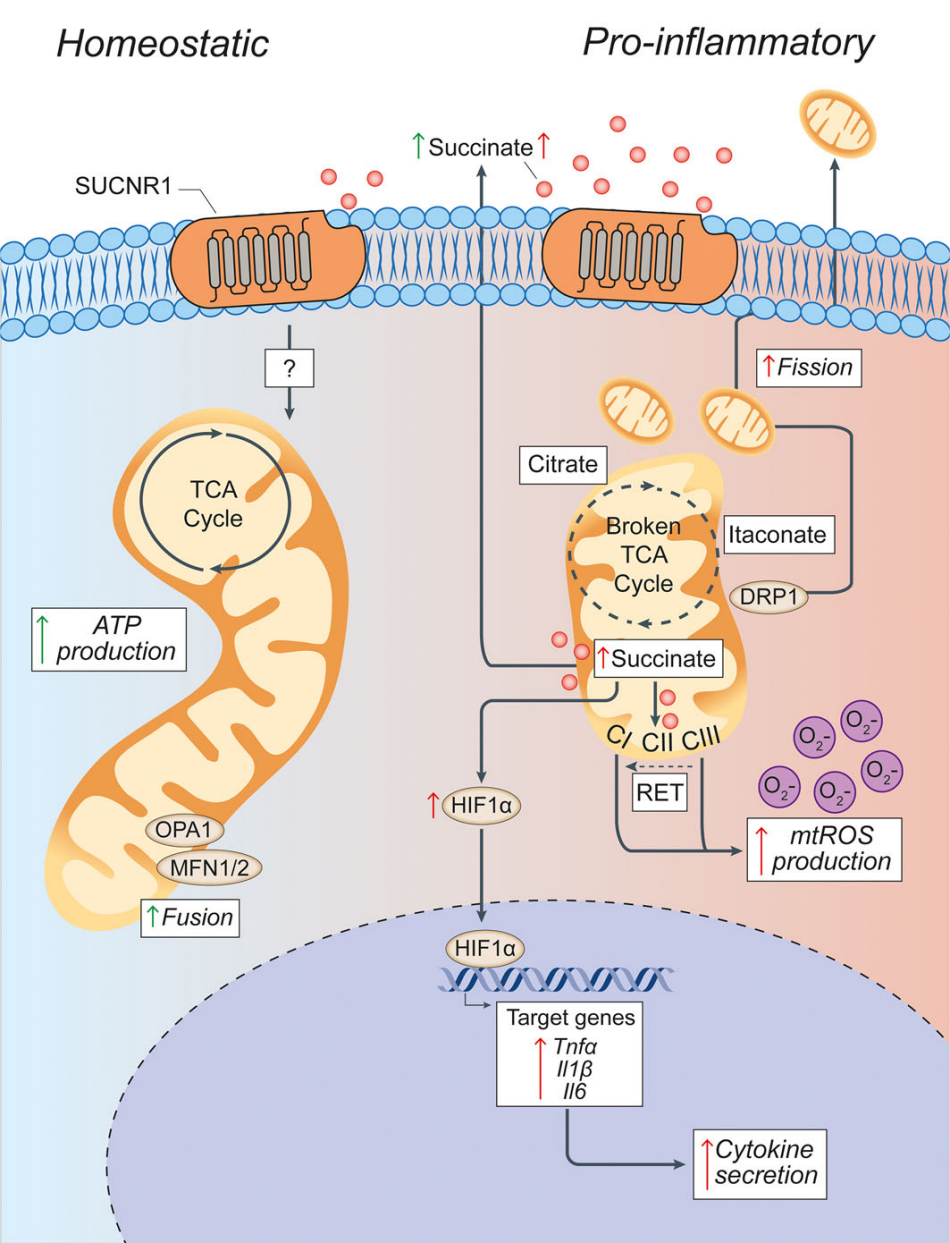
Pro-inflammatory conditions lead to morphological and functional mitochondrial alterations of microglia. Microglia in homeostatic conditions have intact mitochondria with a functioning TCA cycle and conserved fusion of the mitochondrial network through OPA1 and MFN1/2 activities. Succinate signalling through SUCNR1 in homeostasis is presumably low, but its signalling functions in resting microglia are currently undefined. In pro-inflammatory conditions, mitochondria undergo DRP1-mediated fission and fragmentation, and show two breaks in the TCA cycle that lead to the intracellular accumulation of specific metabolites (such as succinate, citrate, and itaconate). Succinate accumulation within mitochondria can drive RET, which produces excessive mtROS through complex I. This mitochondrial dysfunction creates a pseudohypoxic state that leads to the stabilization of HIF1α and enhances cytokine production and secretion. Both fragmented mitochondria and succinate can be released into the extracellular environment where succinate can signal in an autocrine or paracrine manner via SUCNR1, thus modulating both anti-inflammatory and pro-inflammatory effects. Green arrows = anti-inflammatory effects; red arrows = pro-inflammatory effects. SUCNR1, succinate receptor 1; TCA, tricarboxylic acid cycle; OPA1, optic atrophy 1; MFN1/2, mitofusin 1/2; DRP1, dynamin-related protein 1; CI, complex I; CII, complex II; CIII, complex III; mtROS, mitochondrial reactive oxygen species; RET, reverse electron transport; ATP, adenosine triphosphate; HIF1α, hypoxia inducible factor 1 α; mtROS, mitochondrial reactive oxygen species; TNFα, tumor necrosis factor alpha; Il1β, interleukin-1β; Il6, interleukin-6.

Upon pro-inflammatory activation, myeloid cells undergo major changes in the structure and function of their mitochondrial network that are linked with extensive metabolic rewiring (**Figure 3**). Activated amoeboid microglia in demyelinated cerebellar white matter show greater numbers of small and short mitochondria than the ramified microglia in wild-type (WT) mice suggesting a link between mitochondrial fission and microglial cell activation (126). Interestingly, these changes seem to partially differ between macrophages and microglia during neuroinflammation. Using *Ccr2rfp/* ^+^::*Cx3cr1gfp/* ^+^mice, in which tissue-resident microglia and infiltrating monocyte-derived macrophages were labelled with green fluorescent protein and red fluorescent protein respectively, microglia are described to have longer and thinner mitochondria and spherical nuclei than monocyte-derived macrophages in spinal cord tissues at the onset of EAE (126).

Interfering with the mitochondrial dynamics of microglial cells has shown to affect their activation both *in vitro* and *in vivo*. *In vitro* studies suggest that LPS induces mitochondrial fragmentation in microglia *via* DRP1 signalling thus inducing mitochondrial ROS (mtROS) generation (126). Treating microglial cells *in vitro* with mitochondrial fission inhibitor 1 (Mdivi-1), an inhibitor of DRP1, blocks LPS‐induced mitochondrial fragmentation and increases mitochondrial membrane potential, ROS production, and accumulation of intracellular TCA cycle intermediates (e.g., succinate), which is indicative of impaired OXPHOS (63). *In vivo*, microglia isolated from the brains of animals following induction of systemically driven neuroinflammation and con‐current treatment with Mdivi‐1 (from P1 to P3) show attenuated expression of genes related to pro-inflammatory activation (e.g., *iNOS*, *Ptgs2*) suggesting that controlling mitochondrial fission *in vivo* may intrinsically recue microglial activation (63).

Thus, modulating the mitochondrial dynamics of myeloid cells may also have extrinsic effects on neighbouring CNS cells. Indeed, fission events followed by the release of fragmented and dysfunctional microglial mitochondria propagate neuronal death through activation of naïve astrocytes to the neurotoxic A1 state (127). Following from this model, regulating fission and fusion in microglia might reduce the release of dysfunctional extracellular mitochondria, thus lessening the propagation of damage from activated microglia to astrocytes and from astrocytes to neurons. This mechanism is strictly dependent on the altered function of extracellularly released mitochondria, as intact extracellular astrocytic mitochondria instead provide neuroprotection (127–129). Recent work from our group has shown that delivering functional extracellular mitochondria (via extracellular vesicles) is effective in re-establishing normal mitochondrial function in myeloid cells *in vitro* and *in vivo* during neuroinflammation (130). Further studies will be needed to identify the applicability of these findings to the cure of progressive MS and other neurodegenerative disorders (131).

## Mitochondrial Metabolism of Myeloid Cells

Inhibition of mitochondrial respiration drives the pro-inflammatory activity of myeloid cells and prevents their repolarization to an anti-inflammatory phenotype (132). Reduced OXPHOS is linked with major changes to the mitochondrial metabolism that drive diverse intracellular and extracellular signalling functions (**Figure 3**) (24).

The mitochondrial metabolism of myeloid cells has been thoroughly characterized *in vitro* using pro-inflammatory BMDMs. Carbon flux analyses have identified two ‘‘breaks’’ in the TCA cycle: one at the level of isocitrate dehydrogenase (IDH), the enzyme that converts isocitrate to α-ketoglutarate (αKG) and another at the level of succinate dehydrogenase (SDH), which regulates the oxidation of succinate to fumarate (133). These breaks are partially compensated for *via* an enhanced arginosuccinate shunt that feeds into fumarate and malate or *via* increased glutaminolysis. However, they mostly lead to significant metabolic changes that include the decrease of downstream metabolites such as αKG and fumarate, with a concomitant increase of itaconate, citrate, and succinate (24).

The increase of the expression of cis-aconitic acid decarboxylase (CAD) coded by the immunoresponsive gene 1 sustains the production of itaconate from the accumulated isocitrate (134, 135). Itaconate has antimicrobial properties (by inhibiting the citrate-lyase expressed by different bacterial strains) but can also act as an inhibitor of SDH, limit the levels of inflammatory cytokines, and modulate the IkBz-ATF3 inflammatory and nuclear factor erythroid 2-related factor 2 (NRF2) signalling axis (136, 137). Citrate, instead, is used as a precursor for FAS and lipogenesis but also for prostaglandin and nitric oxide (NO) production, thus sustaining the inflammatory activity of myeloid cells (133). Finally, succinate accumulation, which has been attributed to SDH inhibition (136), glutaminolysis replenishing αKG levels (138, 139), and the gamma-aminobutyric acid shunt (140), plays major roles in regulating both extracellular and intracellular inflammatory signalling.

Extracellularly, succinate accumulates in several inflammatory conditions, including in the cerebrospinal fluid, but not in the blood, of mice with chronic EAE (141). Extracellular succinate modulates inflammation *via* binding to its cognate succinate receptor 1 (SUCNR1), thus eliciting complex responses that are tissue- and context-dependent (142). While in DCs the succinate-SUCNR1 axis clearly potentiates the production of pro-inflammatory cytokines (143, 144), its role in the activation of other myeloid cells is still under investigation. On the one hand, a ‘positive-feedback’ mechanism has been described in chronic inflammation, where IL-1β triggers the production and release of succinate from macrophages, which in turn stimulates SUCNR1-expressing cells to maintain chronic inflammation *via* an autocrine and paracrine loop (145, 146). On the other hand, recent evidence suggests that SUCNR1 stimulation prompts an anti-inflammatory phenotype on adipose tissue macrophages and tumour associated macrophages (147, 148).

Intracellularly, succinate regulates signalling mostly by enhancing the pro-inflammatory activity of myeloid cells. Succinate can be transported from the mitochondria *via* the dicarboxylic acid transporters to the cytosol where in excess it impairs prolyl hydroxylase activity by product inhibition leading to HIF-1α stabilization and activation (140). This phenomenon has been defined as pseudohypoxia (149) and leads to the increased production of pro-inflammatory IL-1β (140). The progressive accumulation of succinate also drives the activity of SDH, thus promoting mtROS production (150). This process links mitochondrial activity, cell metabolism, and ROS production and could be key in treating myeloid mediated oxidative injury in chronic neuroinflammation.

## Mitochondrial Function and Oxidative Injury

Recent approaches using toxic RNA sequencing (Tox-seq), which transcriptionally profiles ROS^+^ innate immune cells, has helped to identify neurotoxic CNS innate immune populations in EAE mice (151). When CD11b^+^ cells labelled for ROS production were analysed by single cell RNA sequencing, a specific ROS^+^ microglia cluster is found to display low levels of homeostatic microglia markers (e.g., *P2ry12*, *Sparc*, *Cx3cr1*, and *Tmem119*) but high levels of oxidative stress and pro-inflammatory genes [e.g., *NADPH oxidase subunit 2* (*gp91-phox*), *MhcII*, *Il1b*] (151). In addition, several genes are upregulated in ROS^+^ microglia and macrophages throughout the oxidative stress network, including glutathione transferases (*Gsto2* and *Gstt2*), γ-glutathione peroxidase (*Gpx7*), and the acivicin target genes (*Ggt1* and *Ggt5*) (151). When EAE mice are treated with the compound acivicin, which inhibits the degradation of the antioxidant glutathione by targeting γ-glutamyl transferase, they show decreased oxidative stress and neurodegeneration, even when treatment is started 80 days after disease onset (151). These data suggest that targeting ROS production in innate immune cells is a promising strategy to treat active chronic neuroinflammation, such as that occurring in people with progressive MS.

Under inflammatory conditions, ROS are produced through various mechanisms. Cytosolic ROS are produced by the NADPH oxidase (NOX) family and NO synthases (NOS). Superoxide, OH^−^, and H_2_O_2_ are instead generated in mitochondria at mitochondrial complex I (CI) and III (CIII), which are the main sites of mtROS production (152, 153). Notably, a link exists between these processes where NO regulates the abundance of TCA cycle metabolites (e.g., succinate and itaconate), as well as the catalytic subunits of CI in inflammatory macrophages (154). This oxidative response is counterbalanced by the activity of several enzymes (e.g., catalase, superoxide dismutases, sirtuin 3), coenzymes (e.g., coenzyme Q), and metabolites (e.g., glutathione) with antioxidant activities (155). In addition, transcription factors [e.g., NRF2, Kelch Like ECH Associated Protein 1] control the expression of antioxidant genes (156), while mitochondrial transporter proteins [e.g., uncoupling protein 2] shuttle H^+^ from the intermembrane space to the mitochondrial matrix, leading to decreased membrane potential and mtROS production (157). When these mechanisms are saturated/inhibited, excessive intracellular ROS production can impact ATP synthesis, cytokine production, mtDNA mutation, and post-translational modification of proteins (155). Extracellularly, ROS release from CNS innate immune cells maintains inflammation, while promoting neurodegeneration and demyelination (158). Given the predominant role of mitochondria in ROS production during inflammation, key potential targets for this new approach reside in specific mitochondrial proteins and complexes (**Figure 3**).

CI is a supercomplex of 44 subunits which form three modules: N module (oxidizing NADH and electron input), Q module (electron output to ubiquinone) and P module (proton transport) (159). CI can produce ROS when electrons circulate in the forward or reverse direction, depending on multiple factors that include mitochondrial function, cell metabolism, and cellular type (150). In fact, forward electron transport (FET) can produce proton leak from CI, but a highly reduced pool of coenzyme Q and a large membrane potential can also trigger reverse electron transport (RET) from over-reduced coenzyme Q back to CI, significantly increasing superoxide production (160). During FET, blocking CI with rotenone suppresses electron transport causing electron leak and increased ROS production (161), while rotenone prevents the electron transport back from coenzyme Q and significantly reduces ROS production during RET (152, 160). In microglia and BMDMs, rotenone enhances ROS and pro-inflammatory cytokine production when cells are in a resting state (162, 163), which suggests that ROS results from impaired FET. In line with this, BMDMs displaying a knockout of the CI subunit *Ndufs4* produce more lactate and ROS than WT BMDMs (164). However, in pro-inflammatory myeloid cells excess of the SDH substrate, succinate, stimulates RET and ultimately shifts mitochondrial activity to mtROS production (150). Accordingly, in LPS-stimulated myeloid cells, especially after prolonged treatment (8–24 h), ROS and pro-inflammatory cytokine production are reduced by rotenone, and this effect may be due to decreased RET (152). Targeting this process, without altering the normal function of CI and OXPHOS, as recently shown for ischemia reperfusion injury (165), could be key in treating chronic neuroinflammatory diseases. CIII is another key site of mtROS generation, which can be modulated. Similarly to CI, blocking CIII activity with antimycin A or myxothiazol, for example, in unstimulated BMDMs increases ROS production (166), while in pro-inflammatory BMDMs, blocking CIII reduced NFκB nuclear accumulation as well as ROS and pro-inflammatory cytokine production (167, 168).

Altogether these data suggest that interacting with CI or CIII (dys)functionality may be important to treat CNS inflammatory disorders. Indeed, the use of CI inhibitors such as rapamycin or metformin can inhibit mtROS production by inhibiting CI formation (169) and attenuate the induction of EAE by restricting the infiltration of mononuclear cells into the CNS and down-regulating the expression of proinflammatory cytokines (IFNγ, TNF, IL-6, IL-17, iNOS), cell adhesion molecules, and matrix metalloproteinase 9 (170).

Further studies will be needed to differentiate these effects from the pleiotropic effects that these molecules have on metabolic pathways (e.g., mTOR) and CNS cell types (e.g., oligodendrocytes) (171–174).

## Conclusion

The growing interest in immunometabolism has demonstrated that myeloid cells are well-equipped to quickly adapt to varying environmental challenges, even when access to carbon sources is highly variable, such as in conditions of inflammation. Therapeutically attractive targets have emerged, with preliminary *in vitro* and *in vivo* testing of compounds proving to be promising. Within this framework, two routes to therapeutic relevance have emerged, targeted therapies using small molecules and compounds (175) and non-targeted therapies. In regard to the latter, the use of dietary intervention (e.g., through the ketogenic diet and/or exercise) may hold the most direct and clinically translatable therapeutic approach towards reprogramming myeloid metabolism from harmful to helpful (176). As previously discussed, microglia can utilize ketone bodies as an alternative energy substrate to glucose, and ketosis has been shown to modulate a range of microglial inflammatory processes and reduce Aβ and tau accumulation in AD mice (177). High-fat, low-carbohydrate ketogenic diets are thought to trigger a shift from glucose metabolism towards FA metabolism, which in turn yields increased ketone body concentrations. Interestingly, pre-treatment of mice with a ketogenic diet decreased microglia activation and pro-inflammatory cytokine IL-6, IL-1β and TNF-α levels in the MPTP mouse model of PD (178). Similarly, oral administration of ketone body metabolites such as β-hydroxybutyrate have been shown to reduce microglial inflammation (179), reduce expression of pro-inflammatory cytokines IL-1β, IL-6, CCL2/MCP-1 (180), and inhibit NLRP3 inflammasome activation (181). Metabolic reprogramming has been identified in exercise-related changes in cognition and immune functions, as exercise attenuated age-dependent inflammatory cytokine expression and cognitive decline in mice, while decreasing glycolytic enzymes and increasing phagocytosis in isolated microglia (182).

In conclusion, the impact of metabolism on both immune and non-immune cells in neuroinflammatory conditions has seen a groundswell of interest in the past decade. Ultimately, more work must be done to fully understand how the microenvironment influences the metabolism of cells and how we can better modulate these functions.

## Author Contributions

LP-J designed the review outline, wrote the manuscript, and outlined the figures. CW contributed to sections of the manuscript and designed the figures. RH and GK contributed to sections of the manuscript and provided input for the relative figures. SP critically reviewed manuscript. All authors contributed to the article and approved the submitted version.

## Funding

This work has received support from the National MS Society (USA; grant RG-1802-30200 to SP), the Italian Multiple Sclerosis Association (AISM, grant 2018/R/14 to SP), the United States Department of Defence (DoD) Congressionally Directed Medical Research Programs (CDMRP) (grant MS-140019 to SP), the Bascule Charitable Trust (RG 75149 and RG 98181 to SP), and Wings for Life (RG 104102 to SP). LP-J was supported by a senior research fellowship from FISM - Fondazione Italiana Sclerosi Multipla (cod. 2017/B/5) financed or co financed with the ‘5 per mille’ public funding, and by a Wellcome Trust Clinical Research Career Development Fellowship (RG G105713). RH was supported by the Cambridge Trust (10468562) and is the recipient of a Canadian Scholarship Trust Foundation, an MNI-Cambridge Douglas Avrith Graduate Studentship, and a Rosetrees Trust Studentship (A1850).

## Conflict of Interest

SP is co-founder, CSO, and shareholder (>5%) of CITC Ltd. and iSTEM Therapeutics and co-founder and Non-Executive Director at Asitia Therapeutics; LP-J is a shareholder of CITC Ltd.

The remaining authors declare that the research was conducted in the absence of any commercial or financial relationships that could be construed as a potential conflict of interest.
